# Improvement of Cyclic Void Growth Model for Ultra-Low Cycle Fatigue Prediction of Steel Bridge Piers

**DOI:** 10.3390/ma12101615

**Published:** 2019-05-16

**Authors:** Shuailing Li, Xu Xie, Yanhua Liao

**Affiliations:** College of Civil Engineering and Architecture, Zhejiang University, Hangzhou 310058, China; 11612057@zju.edu.cn (S.L.); 21512196@zju.edu.cn (Y.L.)

**Keywords:** ultra-low cycle fatigue, cyclic void growth model, circular notched specimens, steel bridge piers, high stress triaxiality, moderate stress triaxiality

## Abstract

The cyclic void growth model (CVGM) is a micro-mechanical fracture model that has been used to assess ultra-low cycle fatigue (ULCF) of steel structures in recent years. However, owing to the stress triaxiality range and contingency of experimental results, low goodness of fit is sometimes obtained when calibrating the model damage degradation parameter, resulting in poor prediction. In order to improve the prediction accuracy of the CVGM model, a model parameter calibration method is proposed. In the research presented in this paper, tests were conducted on circular notched specimens that provided different magnitudes of stress triaxiality. The comparative analysis was carried out between experimental results and predicted results. The results indicate that the number of cycles and the equivalent plastic strain to ULCF fracture initiation by the CVGM model calibrated by the proposed method agree well with the experimental results. The proposed parameter calibration method greatly improves prediction accuracy compared to the previous method.

## 1. Introduction

After the 1994 Northridge earthquake in California and the 1995 Kobe earthquake in Japan, it was observed that beam-to-column connections and baseplate connections in steel bridge piers undergo ultra-low cycle fatigue (ULCF) in such events [1,2,3]. This ULCF damage has been shown to cause the progressive collapse of entire structures [4]. The ULCF is characterized by a ductile crack that initiates at the strain concentration position, then this crack expands stably under cyclic loading, and finally, the catastrophic failure occurs in the brittle mode. ULCF has been shown to occur in the areas of strain concentration in steel bridge piers and beam-column connections under cyclic loading [5,6,7]. Unlike traditional high cycle and low cycle fatigue, ULCF experiences large plastic strain amplitude and is usually characterized by few reverse loading cycles (in general less than 100). Therefore, ULCF is of great significance in the seismic design of steel structures.

The Coffin-Manson formula [8,9] has been widely used to predict the low cycle fatigue life of steel structures. In order to predict ULCF life, Ge et al. [10,11,12] introduced a damage index to evaluate the ULCF life in steel bridge piers based on the Coffin-Manson formula and Miner’s rule [13]. Tateishi et al. [14] developed a new fatigue prediction model that can accurately predict the fatigue life of plain material in an extremely large strain range. Xue [15] proposed a uniform expression to predict low cycle fatigue and ULCF by introducing an exponential function and additional material parameters. However, the above empirical models are derived under uniaxial strain conditions and cannot be applied to a multiaxial stress condition. Micro-mechanism-based models have been proposed to solve these problems in recent years and will be discussed below. 

The micro-mechanical models in the literature can be classified into coupled and uncoupled models [16]. The coupled models consider the intercoupling between material constitutive properties and damage. Mear et al. [17] and Leblond et al. [18] modified the Gurson–Tvergaard–Needleman (GTN) model for cyclic loading. Tong et al. [19] proposed a model based on continuous damage mechanics (CDM) to investigate the ULCF behaviour of beam-column connections. However, in coupled models, the model parameter calibration is a complex task due to the interdependency among the parameters as well as the high calculation cost. These shortcomings impede the application of the coupled models. The uncoupled models can be efficient for crack initiation modelling. Since the uncoupled models assume independence between the material constitutive properties and damage, the parameter calibration is simpler compared to that of the coupled models, and the most accurate state-of-the-art constitutive models can be used in the uncoupled models.

Several uncoupled models have been presented in the literature. Kanvinde and Deierlein [20] proposed the cyclic void growth model (CVGM) to predict ULCF life of structural steel based on the Rice-Tracey void growth theory [21]. Owing to some advantages of the CVGM model, such as predicting fracture initiation at the continuous level and being suitable for multiaxial stress condition compared to empirical models, it has received extensive attention in attempting to predict the ULCF damage of steel structural members. Myers et al. [22] and Fell et al. [23] investigated the ULCF fracture initiation of column baseplate connections and steel frame brace components, respectively. Zhou et al. [24] investigated the ULCF behaviour of beam-column connections, and Liao [25] conducted ULCF fracture predictions for welded connection combined square steel pipe column and H-shaped steel beam. In general, the above research results are encouraging, and it is promising to predict the ULCF fracture initiation of steel structural members using the CVGM model. However, the results predicted by the CVGM model largely depend on the calibration values of the model parameters, especially the calibration value of the damage degradation parameter. In some literature [25,26], the calibration results of damage degradation parameters of the CVGM model is rather discrete. Therefore, it is significant to investigate the effect of model parameter calibration methods on the prediction accuracy of CVGM model. Additionally, it has been suggested that the Lode angle parameter should also be accounted for ULCF modelling except for stress triaxiality (*T* = σ_h_/σ_e_), defined as the ratio of the hydrostatic stress (σ_h_) and the Mises stress (σ_e_), especially in the case of low stress triaxiality (T < 0.33) [27,28,29]. However, high stress triaxiality (T > 0.70) and moderate stress triaxiality (0.33 < T < 0.70) are employed in the present study, and the effect of Load angle parameter can be neglected.

In order to improve the prediction accuracy of the CVGM model, tests were conducted on circular notched specimens made of Q345qC steel commonly used in the construction of steel bridges in China. A model parameter calibration method was proposed, and model parameters of the CVGM for Q345qC steel were calibrated at both high and moderate stress triaxiality based on the experimental results and finite element analysis (FEA). Comparisons were made between the experimental results and predicted results to verified the effectiveness of proposed parameter calibration method. Finally, the effect of damage degradation parameter on ULCF life prediction in steel bridge piers was discussed.

## 2. Cyclic Void Growth Model and Parameter Calibration

### 2.1. Cyclic Void Growth Model

Rice and Tracey studied the growth of spherical void in infinitely large ideal elastoplastic materials and deduced the formula of void growth based on the stress triaxiality and equivalent plastic strain [21]:

(1) $$dr/r=C\cdot \exp\left(1.5T\right)d\varepsilon_{eq}^{p}$$

where *r* represents the instantaneous void radius, *C* indicates a material constant, *T* represents the stress triaxiality, and $d\varepsilon_{eq}^{p}=\sqrt{(2/3)d\varepsilon_{ij}^{p}\cdot d\varepsilon_{ij}^{p}}$ represents the equivalent plastic strain increment.

For cyclic loading, the sign of the stress triaxiality, *T*, changes, and Equation (1) can be revised into a more generalized form [20]:

(2) $$dr/r=\mathrm{sign}\left(T\right)\cdot C\exp\left(\left|1.5T\right|\right)d\varepsilon_{eq}^{p}$$

where sign(*T*) represents the sign of the stress triaxiality. It should be noted that if the stress triaxiality is positive, the void will grow, and sign(*T*) = 1. Conversely, if stress triaxiality is negative, the void will shrink, and sign(*T*) = −1.

The void–void interaction is not be considered here. By integrating Equation (2) over the tensile and compressive excursions of loading, the void radius during cyclic loading can be expressed as follows:

(3) $$\ln{\left(r/r_{0}\right)}_{cyclic}=\sum_{tensile\;}C_{1}\int_{\varepsilon_{1}}^{\varepsilon_{2}}\exp\left(\left|1.5T\right|\right)d\varepsilon_{eq}^{p}-\sum_{compressive\;}C_{2}\int_{\varepsilon_{1}}^{\varepsilon_{2}}\exp\left(\left|1.5T\right|\right)d\varepsilon_{eq}^{p}$$

where *ε*_1_ and *ε*_2_ represent the equivalent plastic strains at the beginning and end of the tensile and compressive excursions, respectively. Due to the lack of data to confirm the relative rates of void growth and shrinkage, it is assumed that *C* = *C*_1_ = *C*_2_. The void growth index, *VGI_cyclic_*, for cyclic loading, representing cyclic void growth “demand”, is defined as follows [20]:

(4) $$VGI_{cyclic}=\sum_{tensile\;}\int_{\varepsilon_{1}}^{\varepsilon_{2}}\exp\left(\left|1.5T\right|\right)d\varepsilon_{p}-\sum_{compressive\;}\int_{\varepsilon_{1}}^{\varepsilon_{2}}\exp\left(\left|1.5T\right|\right)d\varepsilon_{eq}^{p}$$

The critical void growth “capacity”, $VGI_{cyclic}^{crit}$, under cyclic loading is determined by a degraded function of its counterpart under monotonic loading, as described Kanvinde and Deierlein [20].

(5) $$VGI_{cyclic}^{crit}=VGI_{mon}^{crit}\exp\left(-\lambda \varepsilon_{p}^{accu}\right)$$

where $VGI_{mon}^{crit}$ represents the monotonic void growth “capacity” [30], *λ* indicates the material damage degradation parameter under cyclic loading and is fitted according to Equation (6), and $\varepsilon_{p}^{accu}$ represents a damage variable that is the cumulative equivalent plastic strain at the beginning of each tensile cycle [20].

(6) $$f=VGI_{cyclic}^{crit}/VGI_{mon}^{crit}=\exp\left(-\lambda \varepsilon_{p}^{accu}\right)$$

where *f* represents the material damage ratio.

ULCF is considered to occur when *VGI_cyclic_* exceeds $VGI_{cyclic}^{crit}$. To quantify the extent of ULCF damage here, a damage index, *D*, has been defined as follows:

(7) $$\left\{ \begin{array}{l} D=\max\left\{D_{n-1},D_{th}\right\}\\ D_{th}=1-\frac{\left(VGI_{cyclic}^{crit}-VGI_{cyclic}\right)}{VGI_{mon}^{crit}} \end{array}\right.$$

where *n* indicates the number of incremental steps during finite element calculation, and *D_th_* represents the value of the damage index calculated by the current incremental step. During the time-history calculation, if *D_th_* exceeds *D_n_*_−1_ of the previous step, then *D* is updated to *D_th_*, otherwise it remains constant. When *D* reaches one, ULCF is considered to occur.

The ULCF fracture initiation is not the failure of a material point but involves a critical volume of material. The characteristic length is defined to reflect the critical volume and can be determined from the microscopic fracture surfaces of specimens by scanning electron microscopy. The proposed characteristic length is commonly determined from two boundary values and a mean value [31]. The lower bound is twice the average diameter of the dimples, the upper bound is the maximum value of a plateau or trough, and the mean value is taken as an average of about ten plateaus or troughs, that is, the most likely value of characteristic length.

### 2.2. Parameter Calibration of Q345qC Steel

#### 2.2.1. Material Property

**Figure 1:**
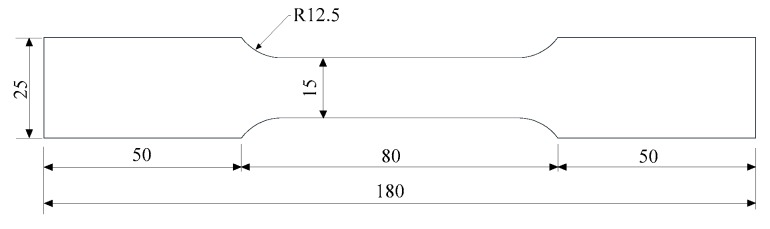
Dimensions of smooth round bar specimens (unit: mm).

Uniaxial tensile tests of three smooth round bar specimens were carried out using MTS 880 (MTS Systems Corporation, Eden Prairie, MN, USA) to obtain mechanical properties of Q345qC steel. The dimensions of the smooth round bar specimens are presented in Figure 1, and the gauge length of the extensometer is 50 mm.

The deformation in the extension gauge was uniform before necking, and the stress–strain curve of the material was fitted according to Equation (8) [32].

(8) $$\sigma =K{\left(\varepsilon_{p}\right)}^{n}$$

where *K* represents the strain hardening coefficient, n indicates the strain hardening index, and represents the plastic strain.

**Figure 2:**
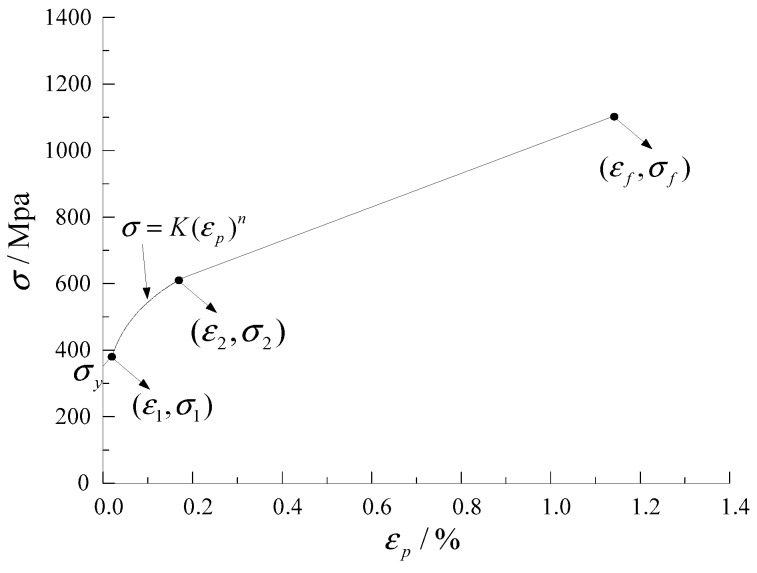
Calibrated true stress–plastic-strain curve.

When the specimen began to neck, the deformation in the extension gauge was concentrated in the necking region. It was assumed that the stress–strain relationship linearly increased from the necking to the fracture. The true stress, σ*_f_*, and true strain, ε*_f_*, of the specimen when fractured can be calculated by Equation (9). The mechanical properties of the material are provided in Table 1. The stress–strain curve of the material is presented in Figure 2, and the key parameters are listed in Table 2.

(9) $$\begin{array}{l} \sigma_{f}=\frac{p_{f}}{\pi d_{f}^{2}/4}\\ \varepsilon_{f}=\mathrm{In}\left[{\left(\frac{d_{0}}{d_{f}}\right)}^{2}\right] \end{array}$$

#### 2.2.2. Calibration of Monotonic Void Growth Capacity

**Figure 3:**
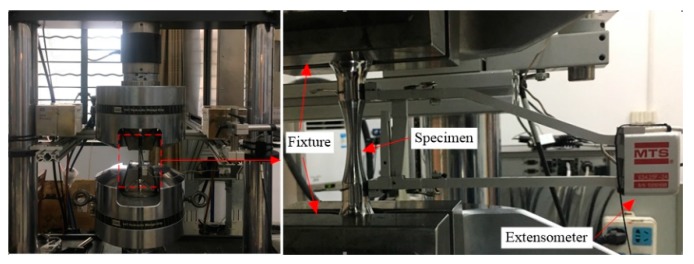
Test setup of notched specimen.

**Figure 4:**
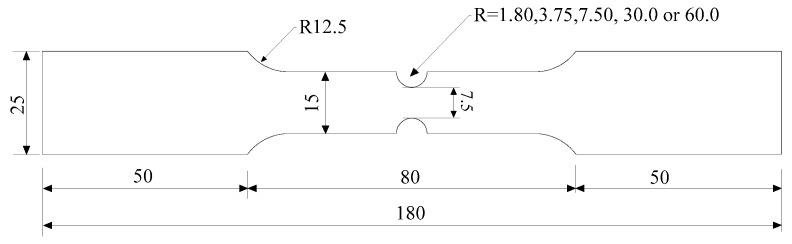
Dimensions of notched specimens for tensile tests (unit: mm).

Uniaxial tensile tests of circular notched specimens were carried out using MTS 880, as presented in Figure 3. The dimensions of the specimens are presented in Figure 4. Loading was applied with strain control, and the gauge length of the extensometer is 50 mm. Since the stress triaxiality may change significantly during loading, a concept of average stress triaxiality was introduced [33], as defined as Equation (10):

(10) $${\bar{T}}_{m}=\frac{1}{\varepsilon_{F}}\int T(\varepsilon_{p})d\varepsilon_{p}$$

where *ε_F_* represents the fracture strain of notched specimens at the instant of crack initiation, and *T*(*ε_p_*) represents the loading history of stress triaxiality obtained by FEA.

**Figure 5:**
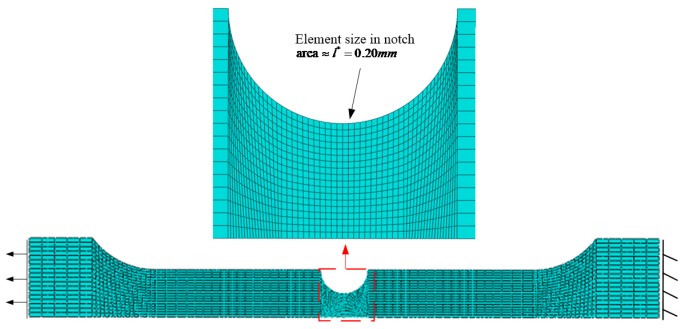
Axisymmetric finite element model of notched tensile specimen (R = 3.75 mm).

The axisymmetry of the specimen geometry and loading procedure allowed for the establishment of a half axisymmetric two-dimensional finite element model of the specimen in ABAQUS 6.14, as presented in Figure 5. The reduced integration element (CAX8R) was adopted, and the element size in the notched area was approximately 0.20 mm in order to be consistent with the characteristic length of Q345qC steel [34].

**Figure 6:**
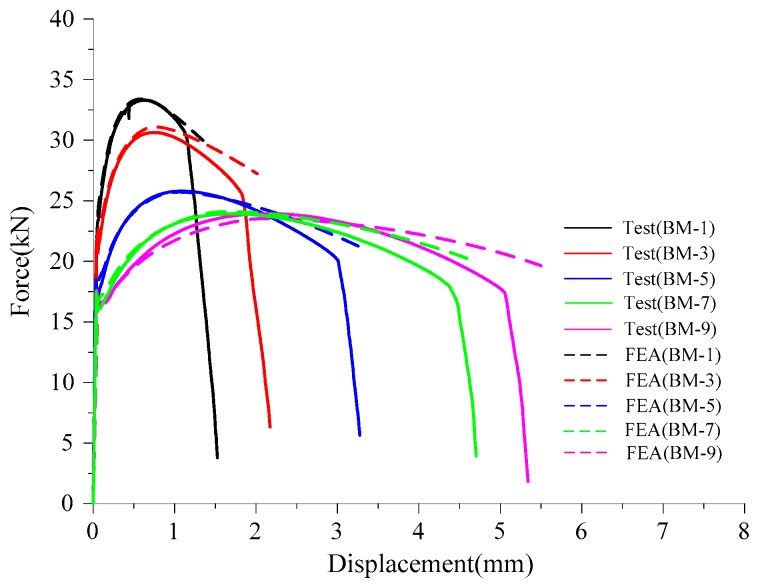
Comparison of force-displacement obtained by tests and FEA under tensile loading.

Figure 6 presents the comparison of force-displacement curves obtained from the tensile tests and FEA, respectively. It can be observed that test curves are in strong agreement with FEA curves. The sudden change in the slope of the force-displacement curve corresponds to the instant of crack initiation [30], and its corresponding displacement, $\Delta_{f}$, is used as the control deformation in the FEA to calculate $VGI_{mon}^{crit}$. The ${\bar{T}}_{m}$ and calibration results of $VGI_{mon}^{crit}$ at the center of net section of specimens are presented in Table 3. The monotonic void growth capacity, $VGI_{mon}^{crit}=2.03$, of Q345qC is smaller than that of Q345B ($VGI_{mon}^{crit}=2.55$) [35]. Thus, it can be deduced that Q345B has greater fracture toughness than that of Q345qC.

#### 2.2.3. Calibration of Damage Degradation Parameter

**Figure 7:**
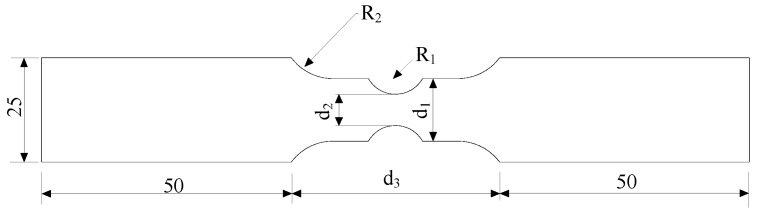
Contour of notched specimens for cyclic tests (unit: mm).

Circular notched specimens were subjected to two types of cyclic loading histories. The contour of the specimens is presented in Figure 7, and the dimensions are provided in Table 4. In one loading history, the applied displacements were cycled between two predetermined values until fracture occurred, which is referred to as the cycle to failure (CTF) loading. In the second loading history, five cycles were applied at specified loading amplitudes, then monotonic tensile pulling to fracture was performed. This second loading history is called the cycle and pull to failure (C-PTF) loading. The loading was controlled by strain, the gauge length of the extensometer of specimens (R_1_ = 60.00 mm) is 50 mm, and that of the other specimens is 12.5 mm. Similarly, the following average stress triaxiality was introduced, defined as Equation (11) below. The loading protocol and average stress triaxiality of specimens for cyclic loading are provided in Table 4.

(11) $${\bar{T}}_{c}=\frac{1}{\varepsilon_{F}}\int \left|T(\varepsilon_{p})\right|d\varepsilon_{p}$$

The FEA of cyclic tests was carried out by ABAQUS 6.14, and the division of elements was similar to that of the tensile loading analysis. The Lemaitre–Chaboche hybrid hardening model [36] was used to simulate the cyclic plastic flow of the material, including isotropic and kinematic hardening. The above calibrated true stress–plastic-strain curve was used to simulate the kinematic behaviour, and the isotropic hardening is described by Equation (12).

(12) $$\sigma^{0}=\sigma_{0}+Q_{\infty }\left[1-\exp\left(-b\varepsilon_{p}\right)\right]$$

where $\sigma_{0}$ represents the size of the initial yielding surface;$Q_{\infty }$ indicates the maximum change value of yielding surface; and *b* denotes the changing rate of yielding surface size as plastic strain develops. The parameters $Q_{\infty }$ and *b* were determined from a trial procedure based on the best fit between the test curves and FEA curves.

**Figure 8:**
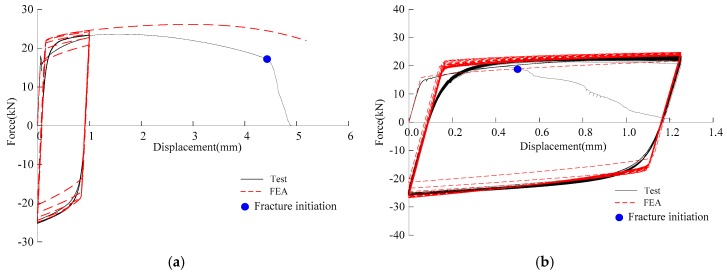

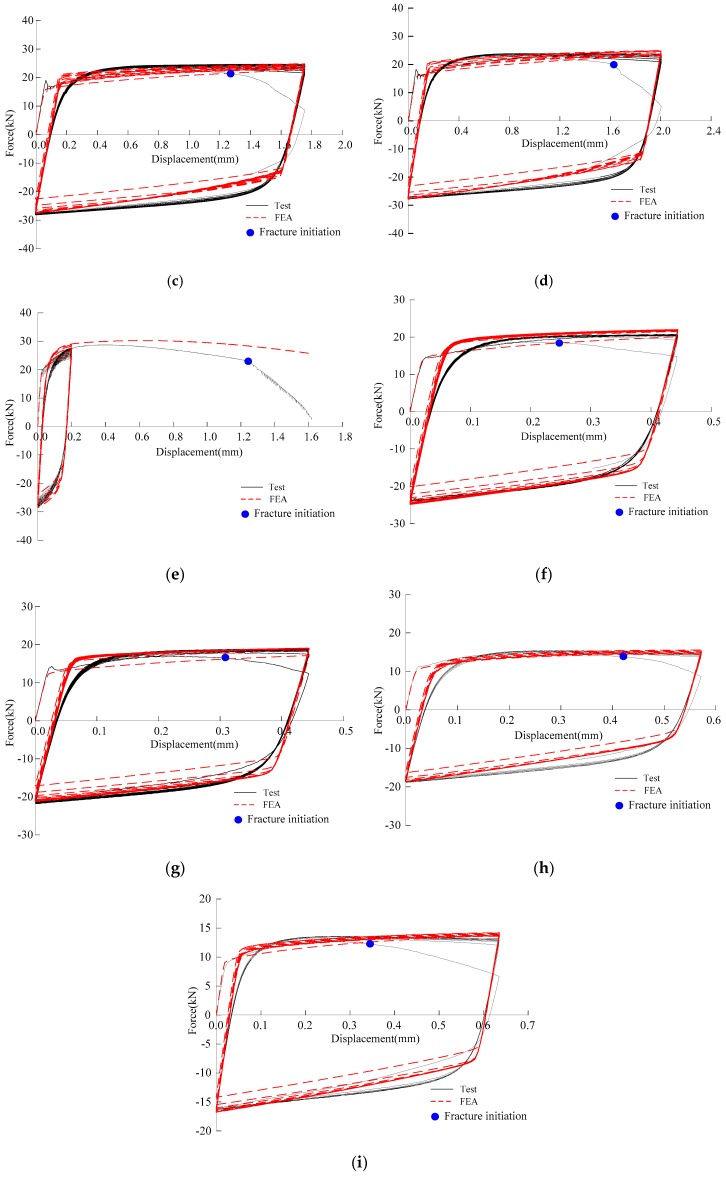
Comparison of force-displacement of specimens obtained by tests and FEA: (**a**) BMC-1; (**b**) BMC-3; (**c**) BMC-5; (**d**) BMC-7; (**e**) BMC-9; (**f**) BMC-11; (**g**) BMC-13; (**h**) BMC-15; (**i**) BMC-17.

Similar to tensile loading, the sudden change in the slope of the last tensile cycle is the instant of crack initiation [20]. Figure 8 presents the force-displacement curves of partial specimens obtained by tests and FEA. It can be observed that the test curves agree with the FEA curves. The other specimens have similar results.

**Figure 9:**
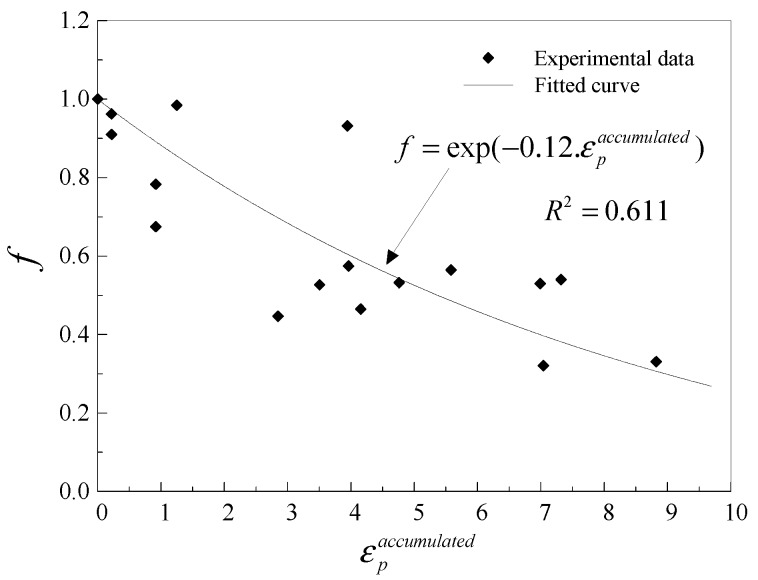
Scatter plot and fitted curve of damage degradation parameter for Q345qC.

The $VGI_{cyclic}^{crit}$ at the center of the section under cyclic loading was calculated, obtaining the material damage ratio, *f*. The size of voids at the critical instant under cyclic loading was smaller than that of monotonic loading, thus *f* was no more than one in theory [31]. The damage ratio of BMC-15 and BMC-17 was greater than one and thus were not used to calibrate the damage degradation parameter. The reason for this may be twofold. First, although the constitutive model used in the FEA has been calibrated, there remains a certain difference compared to the actual constitutive behaviour of the material, and this difference may cause the error of the stress–strain analysis. Second, the calibration of $VGI_{mon}^{crit}$ also lead to an error in calculating the damage ratio. The *f* and $\varepsilon_{p}^{accu}$ were fitted according to the exponential function in Equation (6) to obtain the material damage degradation parameter, *λ*. The experimental data scatter plot and the fitted curve are presented in Figure 9.

**Figure 10:**
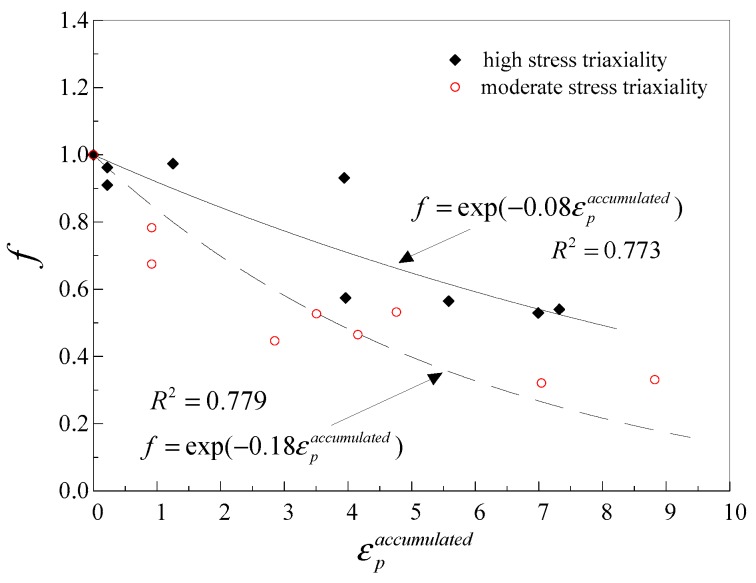
Calibration of damage degradation parameter at high and moderate stress triaxiality.

As shown in Figure 9, the coefficient of determination of the fitted curve is low. This may result in poor ULCF prediction. In order to increase the goodness of fit, the damage degradation parameter was separately calibrated at high and moderate stress triaxialities, as shown in Figure 10, and it can be observed that the experimental data and fitted curves agree well. It can be seen that the damage degradation parameter at moderate stress triaxiality is larger than that at high stress triaxiality. This may be due to the dominant factor in the failure process. Recent experimental and computational studies have shown that micro-void dilation is dominant at high stress triaxiality, and micro-void elongation gradually dominates the failure process of ULCF damage as stress triaxiality decreases [37,38,39].

## 3. ULCF Fracture Initiation Prediction

In order to examine the accuracy of the CVGM model at high and moderate stress triaxiality, cyclic tests were conducted on circular notched specimens providing both high and moderate stress triaxiality. The contour and dimensions of specimens are presented in Figure 7 and Table 5, respectively. Loading was controlled by strain with CTF loading, the gauge length of the extensometer was 50 mm, and the loading strain is provided in Table 5.

**Figure 11:**
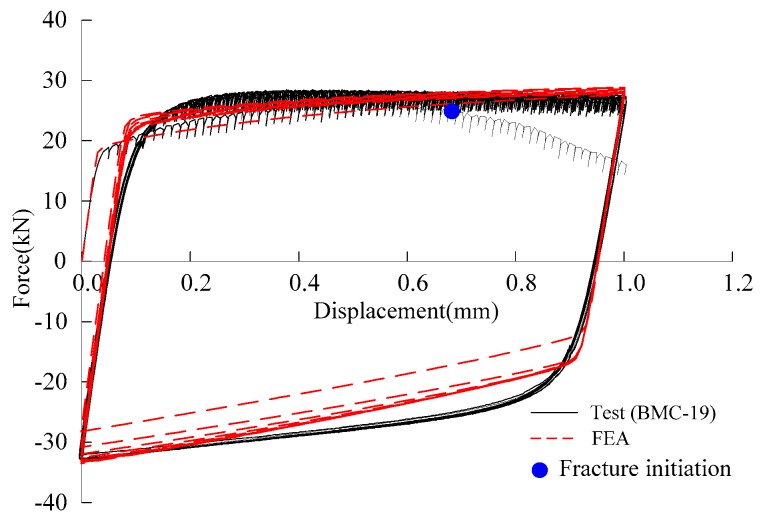
Comparison of force-displacement curve obtained by tests and FEA under cyclic loading.

**Figure 12:**
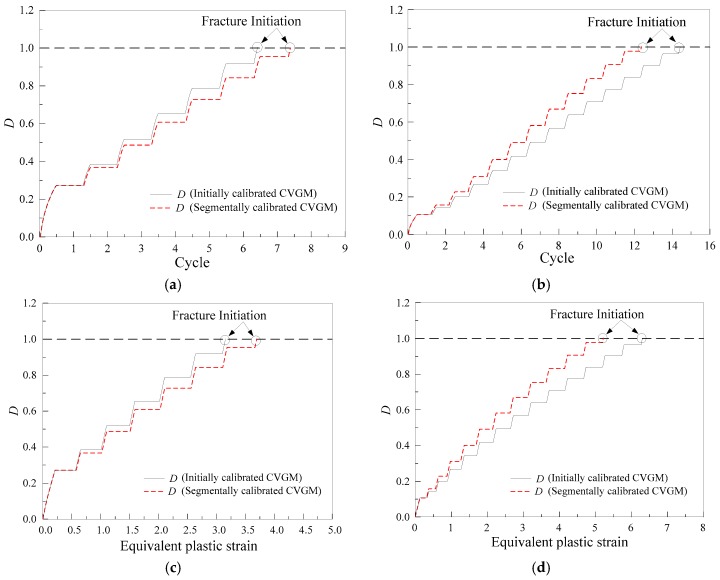
Evolution of damage index Figure: (**a**) BMC-19; (**b**) BMC-28; (**c**) BMC-19; (**d**) BMC-28.

The two-dimensional axisymmetric finite element model was established using ABAQUS 6.14 with the element division similar to that of the aforementioned tensile loading. The Lemaitre-Chaboche hybrid hardening model [36] was used to simulate the cyclic plastic flow of material. Figure 11 presents an example of the force-displacement plot for a specimen, and it can be seen that the test curve is in good agreement with the FEA curve. The development of the damage index, *D*, of partial specimens at the fracture initiation location is presented in Figure 12. ULCF fracture is predicted to occur in the notched specimens when *D* reaches one.

**Figure 13:**
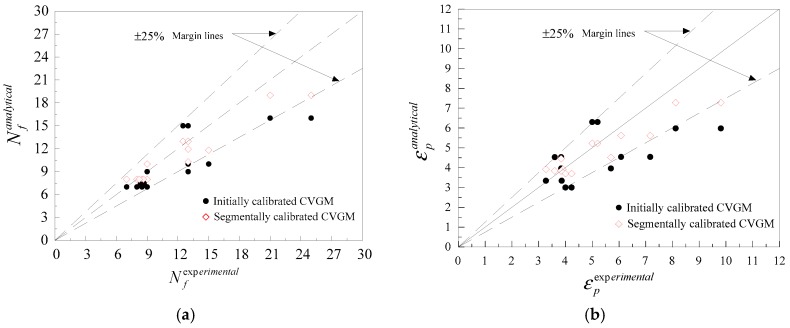
Comparisons of experimental and FEA results: (**a**) Number of cycles to fracture; (**b**) Equivalent plastic strain up to fracture.

Figure 13 presents the comparison of the experimental results and predicted results, including the number of cycles, *N_f_*, and equivalent plastic strain, $\varepsilon_{p}$, to fracture initiation. To evaluate the prediction accuracy of the CVGM model, relative error, γ, is calculated according to Equation (13), and the results are presented in Table 6. The results indicate that the predicted life and equivalent plastic strain by a segmentally calibrated CVGM model is nearer to the experimental results compared to the originally calibrated CVGM model. For a small part of specimens, the errors are larger than or close to 25% between experimental results and predicted results by segmentally calibrated CVGM model, which may be related to some assumptions introduced in the model, but the error is acceptable. In general, the mean value of γ calculated by the segmentally calibrated CVGM model is approximately 50% smaller than that of the initially calibrated CVGM model. Therefore, separately calibrating the CVGM model at high and moderate stress triaxiality greatly improved the prediction accuracy of ULCF.

(13) $$\gamma =\left\{ \begin{array}{l} \frac{\left|\varepsilon_{p}^{analytical}-\varepsilon_{p}^{\exp erimental}\right|}{\varepsilon_{p}^{\exp erimental}}\\ \frac{\left|N_{f}^{analytical}-N_{f}^{\exp erimental}\right|}{N_{f}^{\exp erimental}} \end{array}\right.$$

## 4. Effect of Damage Degradation Parameter

**Figure 14:**
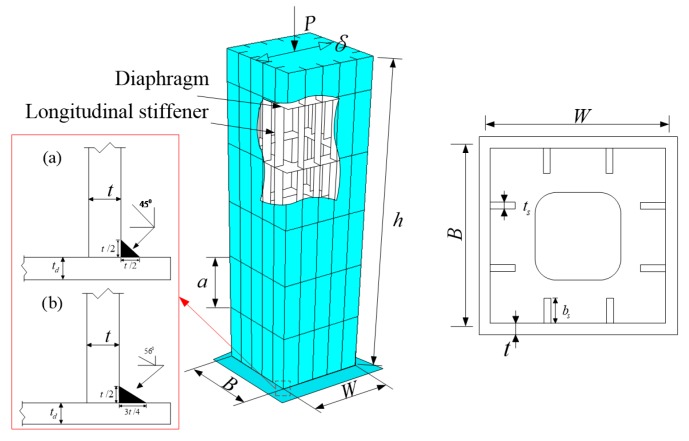
Schematic diagram of single-column steel bridge pier.

**Figure 15:**
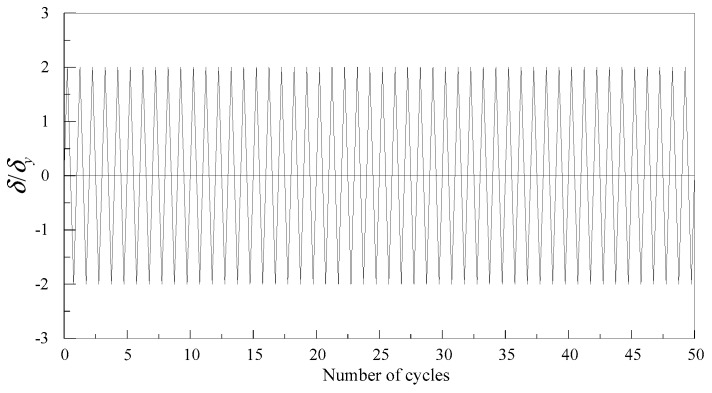
Loading pattern.

The different values of the damage degradation parameter for Q345qC steel were obtained at different stress triaxiality ranges. To investigate the effect of the damage degradation parameter on predicting ULCF fracture initiation of steel structural members, the single-column steel bridge pier was applied as the research object. In this study, two fillet welds were used with groove angles of 45° and 56°, as presented in Figure 14. In Figure 14, *h* represents the height of the pier, *B* represents the width of the flange, *W* represents the width of the web, *t* represents the thickness of the flange and the web, *b_s_* represents the width of the stiffener, *t_s_* represents the thickness of the stiffener, *t_d_* represents the thickness of the diaphragm and baseplate, and *a* represents the spacing of the diaphragms. The geometric dimensions of the steel bridge pier are listed in Table 7. The axial pressure, *P*, and horizontal forced displacement, *δ*, were applied at the top of the pier, and the axial compression ratio is 0.15. The loading pattern is shown in Figure 15, where *δ_y_* is the horizontal yield displacement of the pier.

**Figure 16:**
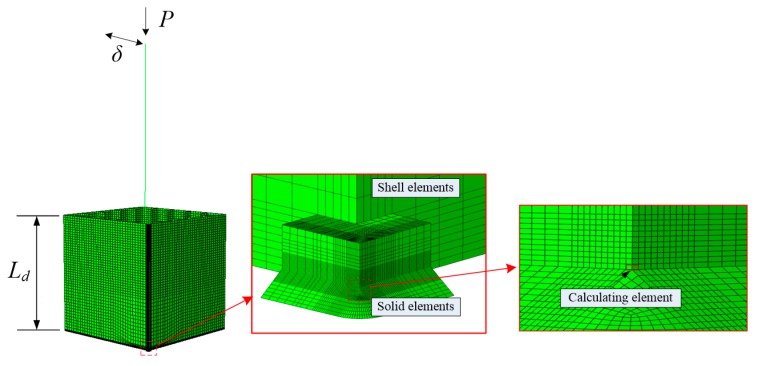
Analytical model of steel bridge piers.

The numerical analyses were conducted using ABAQUS 6.14. Three-dimensional finite element models of the steel bridge pier were established, as shown in Figure 16. The beam element (B31) was employed to simulate the upper part of the steel bridge pier. The lower part of the pier, *L_d_*, was simulated by the shell element (S4R) where the length of *L_d_* was determined according to the empirical formula for calculating the damaged domain [40]. To obtain the stress–strain history at the bottom of the boundary between the flange and web, the solid element (C3D8R) was used. The MPC-beam connection was adopted between the beam and shell element. The shell and solid element were coupled by Shell-Solid. The most dangerous element in the point of connection between the flange and web was considered to be the calculating element, and the size of the calculating element was 0.20 mm, which is consistent with the characteristic length of Q345qC [34]. The Lemaitre–Chaboche hybrid hardening model was used to simulate the cyclic plastic flow of material, and the model parameters used are provided in Table 8 [34]. 

The ULCF life of steel bridge piers with different groove angles was calculated using the above calibrated CVGM model, and the calculation results are shown in Table 9. The predicted ULCF life by segmentally calibrated CVGM model varied by a maximum of 37.5% as compared to the predicted life by initially calibrated CVGM model. Therefore, based on the calculation results, the calibrated damage degradation parameter has a significant influence on the ULCF fracture prediction, and it is important to accurately calibrate the damage degradation parameters under different stress triaxiality ranges. Additionally, due to greater strain concentration, the steel bridge pier with a groove angle of 56° is prone to ULCF damage compared with the steel pier with a groove angle of 45°.

## 5. Conclusions

In the research presented in this paper, Q345qC steel was selected for study as it is commonly used in the construction of steel bridges in China. A model parameter calibration method that separately calibrates the damage degradation parameter at high and moderate stress triaxiality was proposed. The validity of the CVGM model calibrated by the proposed method was verified based on the tests and FEA. The effect of the damage degradation parameter on predicting the ULCF fracture initiation of steel bridge piers was investigated. Based on the conducted studies, the following conclusions can be drawn:

(1) The goodness of fit is improved when the damage degradation parameter is calibrated separately at high and moderate stress triaxiality. It is shown that the predicted number of cycles and equivalent plastic strain to fracture by the segmentally calibrated CVGM model agree well with the experimental results. The proposed parameter calibration method greatly improved the predictive accuracy of the CVGM model compared to the previous method.
(2) From the numerical simulation of steel bridge piers, the value of the damage degradation parameter has a relatively significant influence on the predicted cycles to ULCF fracture initiation. Therefore, it is significant to separately calibrate the damage degradation parameters under different stress triaxiality ranges.
(3) The different values of the damage degradation parameter were obtained at high and moderate stress triaxiality, and it was determined that the value of the damage degradation parameter depends on stress triaxiality. Therefore, it is clear that the relationship between damage degradation parameter and stress triaxiality deserves further study in the future.

In future work, additional experimental data are needed to determine the relationship between damage degradation parameter and stress triaxiality. Additionally, the different values of damage degradation parameter at different levels of stress triaxiality may be related to the fracture mechanism, and as such it requires further research. In general, the proposed parameter calibration method that separately calibrates parameters under different stress triaxiality ranges is promising.

## Figures and Tables

**Table 1:** Mechanical properties of Q345qC steel.

Method	*E* (MPa)	*σ_y_* (MPa)	*σ_u_* (MPa)	*ε_f_*	*σ_f_* (MPa)	A (%)
Mean	198,221	351.10	508.57	1.14	1104.57	40.60
Cov/%	0.83	1.16	1.51	0.75	1.74	3.57

Note: *E* indicates elastic modulus; *σ_y_* and *σ_u_* denote the yield strength and ultimate strength respectively; A represents elongation ratio.

**Table 2:** Constitutive model parameters under tensile loading.

Method	*σ_y_* (MPa)	*ε*_1_	σ_1_ (MPa)	*ε*_2_	σ_2_ (MPa)	*ε*_f_	*σ*_f_ (MPa)	*K* (Mpa)	*n*
Mean	351.10	0.02	364.31	0.17	606.84	1.14	1104.57	906.80	0.22
Cov/%	1.16	3.14	0.39	2.52	0.87	0.75	1.74	0.58	1.20

**Table 3:** Calibration results of monotonic void growth capacity.

Notch Size	No.	$\Delta_{f}$ (mm)	${\bar{T}}_{m}$	$VGI_{mon}^{crit}$
1.80	BM-1	1.16	1.23	2.05
	BM-2	1.20	1.23	2.10
3.75	BM-3	1.64	0.89	1.92
	BM-4	1.80	0.92	2.10
7.50	BM-5	3.18	0.69	2.30
	BM-6	3.00	0.68	2.15
30.0	BM-7	4.35	0.54	1.98
	BM-8	4.36	0.54	1.99
60.0	BM-9	5.05	0.49	1.78
	BM-10	5.32	0.50	1.98
Mean value				2.03
Cov/%				6.56

**Table 4:** Dimensions and loading protocols of circular notched specimens.

R_1_ (mm)	No.	d_1_ (mm)	d_2_ (mm)	d_3_ (mm)	R_2_ (mm)	Loading	Loading Strain	Cycles to Fracture Initiation	${\bar{T}}_{c}$
60.00	BMC-1	15.0	7.50	80.0	12.5	C-PTF	[0, 0.020]	6	0.44
	BMC-2	15.0	7.50	80.0	12.5	C-PTF	[0, 0.020]	6	0.45
	BMC-3	15.0	7.50	80.0	12.5	CTF	[0, 0.025]	24	0.53
	BMC-4	15.0	7.50	80.0	12.5	CTF	[0, 0.025]	20	0.54
	BMC-5	15.0	7.50	80.0	12.5	CTF	[0, 0.035]	10	0.51
	BMC-6	15.0	7.50	80.0	12.5	CTF	[0, 0.035]	11	0.51
	BMC-7	15.0	7.50	80.0	12.5	CTF	[0, 0.040]	7	0.48
	BMC-8	15.0	7.50	80.0	12.5	CTF	[0, 0.040]	8	0.49
1.500	BMC-9	12.5	6.25	50.0	21.0	C-PTF	[0, 0.020]	6	1.17
	BMC-10	12.5	6.25	50.0	21.0	C-PTF	[0, 0.020]	6	1.17
3.125	BMC-11	12.5	6.25	50.0	21.0	CTF	[0, 0.035]	20	1.12
	BMC-12	12.5	6.25	50.0	21.0	CTF	[0, 0.035]	15	1.10
4.500	BMC-13	12.5	6.25	50.0	21.0	CTF	[0, 0.035]	25	0.99
	BMC-14	12.5	6.25	50.0	21.0	CTF	[0, 0.035]	24	0.98
1.500	BMC-15	10.0	5.00	46.0	25.0	CTF	[0, 0.045]	7	1.26
	BMC-16	10.0	5.00	46.0	25.0	CTF	[0, 0.048]	4	1.20
3.500	BMC-17	10.0	5.00	46.0	25.0	CTF	[0, 0.035]	11	0.98
	BMC-18	10.0	5.00	46.0	25.0	CTF	[0, 0.035]	9	0.97

Note: For example, [0, 0.025] refers to the specimen cycled between strain 0 and 0.025.

**Table 5:** Dimensions and experimental results of circular notched specimens.

R_1_ (mm)	No.	d_1_ (mm)	d_2_ (mm)	d_3_ (mm)	R_2_ (mm)	Cycles to Fracture Initiation	Loading Strain	${\bar{T}}_{c}$
7.50	BMC-19	15.0	7.50	50.0	12.5	9	[0, 0.040]	0.85
	BMC-20	15.0	7.50	50.0	12.5	9	[0, 0.040]	0.85
10.0	BMC-21	15.0	7.50	50.0	12.5	9	[0, 0.040]	0.77
	BMC-22	15.0	7.50	50.0	12.5	13	[0, 0.040]	0.80
	BMC-23	15.0	7.50	50.0	12.5	7	[0, 0.045]	0.77
	BMC-24	15.0	7.50	50.0	12.5	8	[0, 0.045]	0.76
15.0	BMC-25	15.0	7.50	50.0	12.5	21	[0, 0.035]	0.72
	BMC-26	15.0	7.50	50.0	12.5	25	[0, 0.035]	0.73
	BMC-27	15.0	7.50	50.0	12.5	13	[0, 0.045]	0.71
	BMC-28	15.0	7.50	50.0	12.5	15	[0, 0.045]	0.72
60.0	BMC-29	15.0	7.50	80.0	12.5	13	[0, 0.030]	0.52
	BMC-30	15.0	7.50	80.0	12.5	13	[0, 0.030]	0.52
	BMC-31	15.0	7.50	80.0	12.5	8	[0, 0.040]	0.50
	BMC-32	15.0	7.50	80.0	12.5	8	[0, 0.040]	0.49

Note: For example, [0, 0.040] refers to a specimen cycled between strain 0 and 0.040 until ULCF failure was observed.

**Table 6:** Relative error of prediction results by CVGM model.

Specimen	Relative Error			
	Life		Equivalent Plastic Strain	
	Initially Calibrated CVGM Model	Segmentally Calibrated CVGM Model	Initially Calibrated CVGM Model	Segmentally Calibrated CVGM Model
BMC-19	0.222	0.111	0.272	0.102
BMC-20	0.222	0.111	0.291	0.125
BMC-21	0.000	0.111	0.031	0.174
BMC-22	0.308	0.231	0.339	0.247
BMC-23	0.000	0.143	0.021	0.199
BMC-24	0.125	0.000	0.135	0.016
BMC-25	0.238	0.095	0.264	0.103
BMC-26	0.360	0.240	0.390	0.258
BMC-27	0.231	0.077	0.253	0.076
BMC-28	0.333	0.200	0.367	0.216
BMC-29	0.154	0.000	0.219	0.010
BMC-30	0.154	0.000	0.212	0.004
BMC-31	0.125	0.000	0.258	0.067
BMC-32	0.125	0.000	0.180	0.000
Mean value	0.186	0.094	0.231	0.114

**Table 7:** Geometric dimensions of steel bridge piers.

*h*/mm	*B*/mm	*W*/mm	*t*/mm	*a*/mm	*b_s_*/mm	*t_s_*/mm	*t_d_*/mm
2.5	825	825	20	412	81	10	15

**Table 8:** Lemaitre–Chaboche hybrid hardening model parameters of Q345qC steel [34].

Material	σ|0 (MPa)	Q∞ (MPa)	b	C1 (MPa)	γ1	C2 (MPa)	γ2	C3 (MPa)	γ3
Base metal	351.10	13.2	0.60	44373.7	523.8	9346.6	120.2	946.1	18.7
Weld deposit metal	428.45	17.4	0.40	12752.3	160.0	1111.2	160.0	630.5	26.0

Note: The meaning of parameters can be found in the literature [34].

**Table 9:** Calculation results of ULCF life of steel bridge piers.

Groove Angle	Predicted Cycles to ULCF Fracture Initiation		$\frac{\left|N_{f1}-N_{f2}\right|}{N_{f1}}$	${\bar{T}}_{c}$
	*N_f_*_1_	*N_f_*_2_		
45°	45	29	35.5%	0.68
56°	8	11	37.5%	0.94

Note: *N_f_*_1_ indicates the cycles to ULCF by initially calibrated CVGM model; *N_f_*_2_ indicates the cycles to ULCF by segmentally calibrated CVGM model.
